# The Rheb-mTORC1 Coordinates Cell Cycle Progression and Endoreplication in *Bombyx mori*

**DOI:** 10.3390/insects16070647

**Published:** 2025-06-20

**Authors:** Zhangchen Tang, Huawei Liu, Qingsong Liu, Xin Tang, Jiahui Xu, Gan Luo, Qingyou Xia, Ping Zhao

**Affiliations:** 1 Integrative Science Center of Germplasm Creation in Western China (Chongqing) Science City, Biological Science Research Center, Southwest University, Chongqing 400715, China; 2 Key Laboratory for Germplasm Creation in Upper Reaches of the Yangtze River, Ministry of Agriculture and Rural Affairs, Chongqing 400715, China; 3 Chongqing Key Laboratory of Chinese Medicine & Health Science, Chongqing Academy of Chinese Materia Medica, Chongqing College of Traditional Chinese Medicine, Chongqing 400065, China; 4 Wanzhou Center for Animal Husbandry Industry Development of Chongqing, Chongqing 404100, China

**Keywords:** *Bombyx mori*, cell cycle, endoreplication, mTORC1, Rheb, silk gland

## Abstract

The mechanistic target of the Rapamycin complex 1 (mTORC1) signaling pathway plays a pivotal role in regulating crucial life processes, including cell growth and proliferation, by sensing and integrating various signals, such as growth factors, energy status, and amino acids. Our previous studies showed that activation of the mTORC1 signaling pathway enhances silk protein synthesis and silk gland size. Here, the potential of the molecular mechanism mTORC1 to regulate the growth and development of silk gland cells was investigated. Inhibiting mTORC1 with rapamycin decreased proliferation in the *Bombyx mori* embryonic (BmE) cells and endoreplication in silk gland cells, reducing CyclinB and CyclinE protein levels and DNA content, and arresting the BmE cell cycle at G2/M. Conversely, the overexpression of *Ras* homolog enriched in brain (*Rheb*) led to increased proliferation of BmE cells and endoreplication in silk gland cells, as well as a significant elevation in DNA content. This study provides a molecular explanation for the increase in silk protein synthesis and silk gland length through the activation of mTORC1, thereby refining the regulatory network of the silkworm endoreplication and providing new molecular targets for breeding high-yield varieties of *Bombyx mori*.

## Simple Summary

The silkworm (*Bombyx mori*) is an important insect for the economy, which produces more than 78% of worldwide raw silk. The silk gland serves as the site for the synthesis and secretion of silk proteins; thus, comprehending the molecular mechanisms that control its growth could significantly enhance silk production in silkworms. In this study, we focus on BmE cells and silk glands to systematically investigate the molecular regulatory mechanisms of the mTORC1 signaling pathway in the cell cycle progression of *Bombyx mori*. The study reveals that Rheb-mTORC1 regulates the proliferation and cell cycle progression of BmE cells by modulating CyclinB and CyclinE levels and drives endoreplication in silkworm posterior silk gland cells, thereby increasing their DNA content and enhancing proliferation efficiency.

## 1. Introduction

Silkworm (*Bombyx mori*) is a commercially significant insect due to its ability to produce silk utilized in textiles, cosmetics, and biomaterials [1]. The silk gland serves as the site for the synthesis and secretion of silk proteins; thus, comprehending the molecular mechanisms that control its growth could significantly enhance silk production in *Bombyx mori*. The cell cycle of the silkworm features multiple tissue-specific cell divisions [2,3,4]. Distinct from other mitotic cells, the silk gland also contains endoreplication cells [5].

Endoreplication in silk glands resembles that in *Drosophila* salivary glands, where only the alternation between the G1 and S phases occurs without nucleoplasmic division. This process leads to DNA replication and somatic cell enlargement [6,7]. The transition from mitosis to endoreplication requires the interaction of various signaling pathways with multiple regulators. Genes enriched in silk glands associated with the mTOR, InR, and PI3K/Akt pathways suggest a significant role in silk gland development [8,9,10,11]. Previous research on the mechanisms supporting endoreplication in silk gland cells has primarily focused on identifying genes that sustain endoreplication, such as Ras1 [12], Myc [13], Fzr [14], BmSu(H) [15], and BmE2F1 [16]. In *Bombyx mori,* a posterior silk gland (PSG) specific Fzr mutation has been shown to impede endoreplication progression, causing an expression dysregulation of cyclin proteins and DNA replication-related regulators [17]. BmE2F1 regulates endoreplication in silk gland cells of silkworms through dual mechanisms: firstly, by enhancing the formation of the DNA replication complex; and secondly, by facilitating the cells’ entry into the S phase [16]. However, the regulatory mechanisms underlying endoreplication in the silk gland cells remain poorly characterized and necessitate further comprehensive investigation.

mTOR, an evolutionarily conserved kinase, assembles into the mTORC1 through interactions with regulatory subunits [18,19,20]. The mTORC1 signaling pathway, ubiquitously conserved across eukaryotes, orchestrates cellular growth and proliferation primarily via phosphorylation of two principal downstream effectors: p70 S6 kinase (S6K) and eukaryotic translation initiation factor 4E-binding protein 1 (4EBP1) [21,22,23]. The mechanistic Target of Rapamycin Complex 1 (mTORC1) is a highly conserved signaling hub that integrates environmental and intracellular cues to regulate cell growth, metabolism, and autophagy. The silkworm mTORC1 complex comprises orthologs of canonical components such as BmTOR, BmRaptor, and BmLST8, and plays pivotal roles in DNA synthesis, protein synthesis, and silk gland development [24,25,26]. *Drosophila* mutants deficient in *dS6K*, a downstream target protein of mTORC1, exhibit pronounced developmental delays and severe body size reduction despite the number of cells remaining unchanged [27,28]. Overexpression of *dRheb*, a mTORC1 activator, leads to tissue overgrowth and increased cell size throughout the organism when occurring at the individual level, whereas it results in an accumulation of cells in the S phase when occurring at the cellular level [29]. Our previous study found that overexpression of *Rheb* in the PSG of silkworm resulted in a significant increase in cocoon shell weight and cocoon shell rate, with the silk glands appearing notably enlarged in this strain; however, the exact molecular mechanism remains unclear [25,26].

In China, silkworm is an important insect for the economy, which produces more than 78% of worldwide raw silk [30]. Moreover, silkworm is also an important model insect of *Lepidoptera*. Studying the mTORC1 signaling pathway in the silk gland is beneficial to elucidate the regulatory mechanisms of endoreplication in *Lepidoptera* insects and obtain high-yield silkworm varieties to assist the development of the sericulture industry [4,10,31]. In this study, we focus on BmE cells and silk glands to systematically investigate the molecular regulatory mechanisms of the mTORC1 signaling pathway in the cell cycle progression of *Bombyx mori*. Through inhibition of the mTORC1 signaling pathway by rapamycin, we found that inactivation of this pathway significantly disrupts cell cycle homeostasis and inhibits endoreplication in silk gland cells. Further studies involving the overexpression of the *Rheb* at both cellular and organismal levels revealed that *Rheb* enhances the activation state of the mTORC1 signaling pathway and significantly promotes DNA replication efficiency and cell proliferation.

## 2. Materials and Methods

### 2.1. Insects and Cells

The D9L strain was provided by the Biological Science Research Center of Southwest University. The transgenic OV-Rheb strain was constructed in our previous work [26]. Silkworm larvae were reared on fresh mulberry leaves at 27–28 °C, under a photoperiod of 12 h light, 12 h dark, and 85% relative humidity in an artificial climate box.

BmE cells were cultured and maintained in Grace (Gibco, Waltham, MA, USA) medium supplemented with 10% (*v*/*v*) fetal bovine serum (Gibco, Waltham, MA, USA) and 1% penicillin/streptomycin (Gibco, Waltham, MA, USA).

### 2.2. Rapamycin Treatment

Rapamycin (Beyotime, Shanghai, China) was diluted with BmE cell culture medium, and then Rapamycin-BmE cell culture medium was configured with different concentration gradients. Rapamycin was added into the cell plates with spread cells and incubated in the cell culture incubator at 27 °C. Samples from each group were collected in triplicate at 12, 24, 36 h, for total RNA and protein extraction.

The epidermis of silkworms was first disinfected with 75% ethanol. Subsequently, silk glands were dissected and washed with PBS and transferred to 12-well cell plates containing BmE complete medium with DMSO or Rapamycin at 27 °C. Samples from each group were collected in triplicate at 12, 24, and 36 h, for total RNA and protein extraction.

### 2.3. CCK-8 Cell Proliferation Viability Assay

The well-growing cells were diluted to 3000 cells in 100 μL. The diluted cell suspension was transferred to 96-well cell culture plates and incubated at 27 °C for 12 h. We added 10 μL of pre-thawed CCK-8 (Beyotime, Shanghai, China) to the medium, and the plates were lightly mixed and incubated at 27 °C for 0–48 h under dark conditions. Six samples were set up in each group, and 3 independent biological experiments were repeated. The absorbance value at 450 nm was determined by a microplate reader.

### 2.4. RNA Extraction and Quantitative Real-Time PCR (qPCR) Analysis

Total RNA was extracted from silk glands and BmE cells using TRIzol reagent (Invitrogen, Waltham, MA, USA) according to the manufacturer’s instructions, and the concentration was determined using a spectrophotometer (NanoDrop, 2000, Thermo Fisher Scientific, Waltham, MA, USA). The RNA was reverse transcribed into cDNA using a reverse transcription kit (Takara, Kusatsu, Japan). Finally, the RNA was analyzed using NovoStart^®^SYBR qPCR SuperMix-plus (Novoprotein, Suzhou, China) on a qTOWER2.2 real-time quantitative PCR machine (Analytikjena Biometra, Göttingen, Germany). The eukaryotic translation initiation factor 4A (BmeIF-4α) was regarded as the internal reference gene. All primers used in the qPCR process are listed in Table S1.

### 2.5. Western Blot (WB)

Total proteins from cells and silk glands were extracted using RIPA (Beyotime, Shanghai, China) and lithium thiocyanate (LiSCN), respectively. Protein concentrations were quantified using the Bradford Protein Assay Kit (Beyotime, Shanghai, China). The protein mixture was separated using a FuturePAGE™ 12% 12 wells pre-prepared gel (ACE, Shanghai, China) transferred to a PVDF membrane. The membrane was blocked with TBST containing 5% difco skimmed milk powder for 1 h, and then incubated with phospho-S6K (1:5000, Cell Signaling Technology, Danvers, MA, USA), CyclinB (1:5000, ChemCruz™, Heidelberg, Germany), CyclinE (1:5000, ChemCruz™, Heidelberg, Germany), and tubulin (1:20,000, Beyotime, Shanghai, China) at 37 °C in TBST for 1 h and washed three times with TBST every 10 min. Next, the membrane was incubated with horseradish peroxidase-labeled goat anti-rabbit IgG (1:20,000, Beyotime, Shanghai, China) or goat anti-mouse IgG (1:20,000, Beyotime, Shanghai, China) at 37 °C in TBST for 1 h and washed three times with TBST every 10 min. Finally, the signals were detected using a Super Signal West Femoto detection system (Thermo Fisher Scientific, Waltham, MA, USA) and scanned using a Chemi-scope 3400 mini-instrument (Clinx Scientific, Shanghai, China). Tubulin was used as the internal control.

### 2.6. Cell Cycle Analysis

After treatment with Rapamycin, cells were lightly washed with 500 μL PBS and then blown down with 1 mL PBS, and then centrifuged at 800× *g* for 5 min to precipitate the cells. Then cells were fixed in cold 70% alcohol at 4 °C overnight. The fixed cells were washed with cold PBS and then stained in a propidium iodide solution (Yeasen, Shanghai, China) containing 0.1 mg/mL RNase A (Yeasen, Shanghai, China) for 1 h in the dark at 27 °C. Cell cycle analysis was performed using the CytoFlex flow cytometer (Beckman, Brea, CA, USA) and graphical analysis was performed using ModFit 5 analysis software.

### 2.7. Ethynyl-2-Deoxyuridine (EdU) Assay

The regulatory role of the mTORC1 signaling pathway in cell proliferation in *Bombyx mori* was assessed using the EdU assay (Beyotime, Shanghai, China) according to the manufacturer’s instructions. The cells were first transfected in 12-well cell culture plates for 48 h or after treatment with Rapamycin of cells and silk glands, the medium was removed and 500 μL of warmed cell culture medium (containing 1 μL of EdU (10 nM)) was added at 37 °C. And the cells were incubated in a cell culture incubator at 27 °C for 2–4 h. The cells were then fixed in 4% paraformaldehyde at room temperature for 15–30 min and incubated with Immunol Staining Wash Buffer (Beyotime, Shanghai, China) for 15 min at room temperature. After washing with Immunol Staining Blocking Buffer (Beyotime, Shanghai, China) 3 times, the Click reaction solution was added to each well, and incubated for 30 min in the dark. _Then_ the cells were stained with 200 μL of DAPI (Beyotime, Shanghai, China) for 10 min, and were observed by fluorescence microscope (Olympus, Tokyo, Japan). The percentage of EdU-positive cells was calculated from 3 random areas.

### 2.8. DNA Content Analysis

The DNA content of both cells and silk glands was assessed using The E.Z.N.A.^®^ Tissue DNA Kit (Omega, Guangzhou, China) according to the manufacturer’s instructions. After washing the samples with PBS, we added 200 μL of PBS, 25 μL of OB, and 4 μL of RNase A following transfection or Rapamycin treatment. We then added 220 μL of BL buffer and incubated the samples at 70 °C for 10 min. After adding 220 μL of 100% ethanol and mixing thoroughly, we transferred the samples to HiBind mini columns and centrifuged them at 12,000 rpm for 1 min. We discarded the supernatant, added 500 μL of HBC, and centrifuged them again at 12,000 rpm for 1 min. Following the addition of 700 μL of DNA Wash Buffer and another centrifugation at 12,000 rpm for 1 min, we discarded the supernatant once more and performed an additional 2 min centrifugation at 12,000 rpm. We then inserted the HiBind mini columns into RNase-free centrifuge tubes, added 100–200 μL of warmed EB, allowed the samples to stand for 2 min, and centrifuged at 12,000 rpm for 2 min. Finally, we aspirated the filtrate, performed a second 2 min centrifugation at 12,000 rpm, measured the DNA concentration using a spectrophotometer (NanoDrop 2000), and stored the samples at −20 °C.

### 2.9. Statistical Analysis

All statistical analyses were performed using GraphPad Prism 8.2 software (GraphPad Software, San Diego, CA, USA), and the values are presented as the means ± SEM. Statistical differences between two groups were statistically analyzed using a two-tailed unpaired Student’s *t*-test, and significant differences were defined as * *p* < 0.05, ** *p* < 0.01, *** *p* < 0.001.

## 3. Results

### 3.1. Exploration of the mTORC1 Signaling Pathway Inhibitor Rapamycin

**Figure 1:**
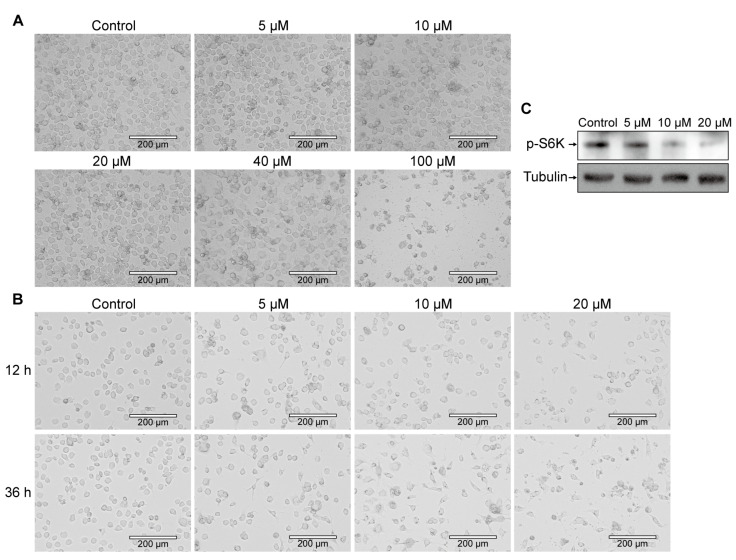
Effects of different concentrations of Rapamycin treatment on BmE cells. (**A**) Morphological changes in BmE cells after 24 h of Rapamycin treatment. Scale bar is 400 μm. (**B**) Morphological changes in BmE cells after 12 h and 36 h of Rapamycin treatment. Scale bar is 400 μm. (**C**) The phosphorylation level of S6K in BmE cells was detected by WB after 12 h of Rapamycin treatment.

To investigate the impact of the mTORC1 signaling pathway on silkworm cell cycle processes, we utilized Rapamycin, a specific inhibitor of mTORC, to treat the BmE cell. To establish the optimal treatment content, we initially assessed the viability of BmE cells via morphological analysis after exposure to various concentrations of Rapamycin for 24 h. The results showed that high concentrations of Rapamycin (40 μM and 100 μM) significantly reduced the number of viable BmE cells and induced substantial cell debris formation compared to untreated controls. In contrast, lower concentrations of Rapamycin (5 μM, 10 μM, and 20 μM) did not significantly alter the morphology of BmE cells, although cell clustering was observed (Figure 1A).

Subsequently, we further explored the optimal concentration and duration of Rapamycin treatment using these lower concentrations (Figure 1B). Simultaneously, we assessed the phosphorylation level of S6K, an indicator of mTORC1 signaling pathway activity, after a 12 h treatment (Figure 1C). Finally, we determined that 12 h treatment with 10 μM Rapamycin was the optimal condition for subsequent experiments, and all subsequent cell treatment assays were conducted under these standardized conditions.

### 3.2. Inhibition of mTORC1 Signaling Pathway Arrests the BmE Cell Cycle at the G2/M Phase and Reduces Proliferation

**Figure 2:**
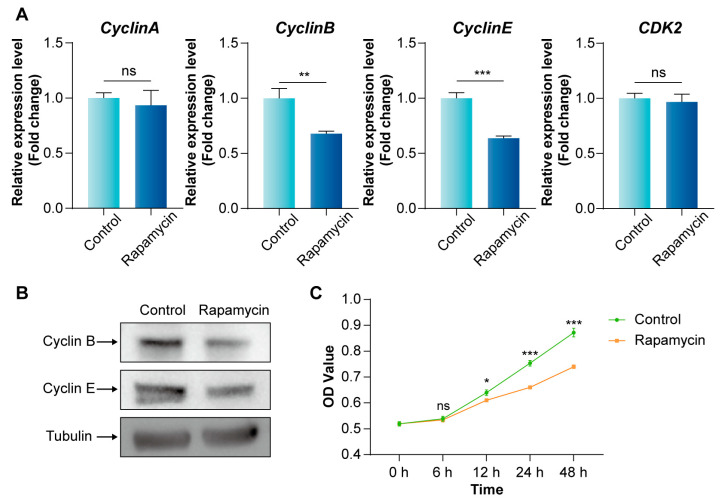
Effect of inhibiting mTORC1 signaling pathway on BmE cell cycle. (**A**) The expression level changes in cell cycle-related genes after Rapamycin treatment were measured by qPCR. Error bars indicate SEM (n = 3). (**B**) The protein abundances of CyclinB and CyclinE after Rapamycin treatment detected by Western blot. (**C**) Effect of Rapamycin on proliferative viability of BmE cells. Error bars indicate SEM (n = 6). Statistically significant differences were assessed using Student’s *t*-test (ns > 0.05, * *p* < 0.05, ** *p* < 0.01 and *** *p* < 0.001).

To explore the role of the mTORC1 signaling pathway in the cell cycle of Bme cells, qPCR and WB were used to detect the changes in cell cycle-related genes and proteins in BmE cells treated with 10 μM Rapamycin for 12 h. The results showed that the expressions of *CyclinB* and *CyclinE* were significantly down-regulated, while the expressions of *CyclinA* and *CDK2* were not significantly changed (Figure 2A). Concurrently, there was a noticeable decrease in the protein levels of CyclinB and CyclinE (Figure 2B). In addition, we also found that the proliferation viability of BmE cells decreased more significantly with the prolongation of Rapamycin treatment time (Figure 2C). These observations suggest that inhibiting the activity of mTORC1 signaling pathway hinders the cycle progression of the BmE cells.

**Figure 3:**
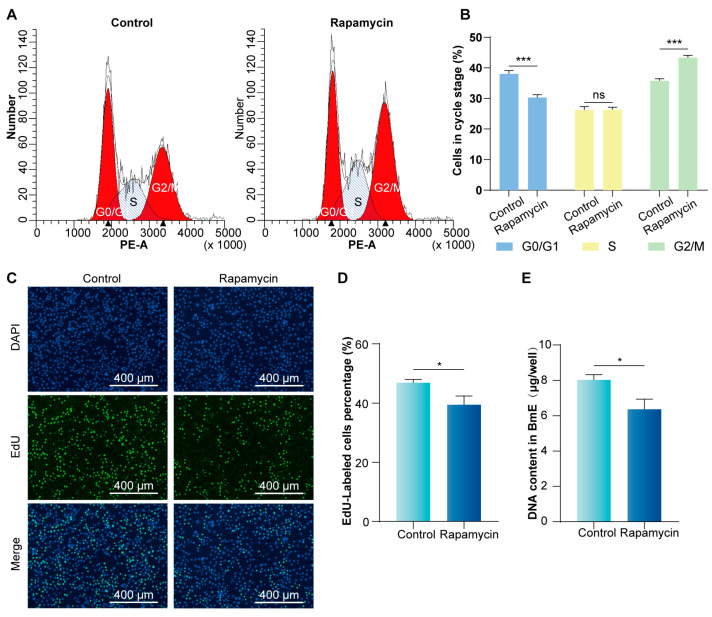
mTORC1 signaling pathway activity affects BmE cell cycle and cell proliferation. (**A**,**B**) Flow cytometry analysis of BmE cell cycle. (**C**) Effect of inhibiting mTORC1 signaling pathway activity on cell proliferation. Proliferating cells were labeled with EdU (green fluorescence). Cell nuclei were stained with DAPI (blue fluorescence). Control, negative control with DMSO addition. Scale bar is 400 μm. (**D**) Statistical analysis of the proportion of EdU-positive cells. The number of cells was calculated from three randomized regions. (**E**) DNA content analysis. DNA extraction was performed after treatment of cells in 6-well cell plates. Error bars indicate SEM (n = 3). Statistically significant differences were assessed using Student’s *t*-test (ns > 0.05, * *p* < 0.05, *** *p* < 0.001).

We further evaluated the role of the mTORC1 signaling pathway in regulating the cell cycle of silkworm by flow cytometry. Flow cytometry analysis showed that the proportion of cells in the G2/M phase increased, while the proportion in the G0/G1 phase decreased after treatment with Rapamycin (Figure 3A,B), indicating that inhibition of mTORC1 activity resulted in cell cycle arrest in the G2/M phase.

Furthermore, changes in cell cycle progression influenced cell proliferation. The EdU assay revealed a 39.50% reduction in the proportion of fluorescently labeled cells in the treated group (Figure 3C,D). Moreover, the DNA content was significantly lower in the treated group (Figure 3E). These findings confirm that the mTORC1 signaling pathway is crucial for regulating the BmE cell cycle, particularly in controlling cells entry into the division phase.

### 3.3. Enhanced mTORC1 Signaling Pathway Activity via Rheb Overexpression Promotes BmE Cell Proliferation and S Phase Progression

**Figure 4:**
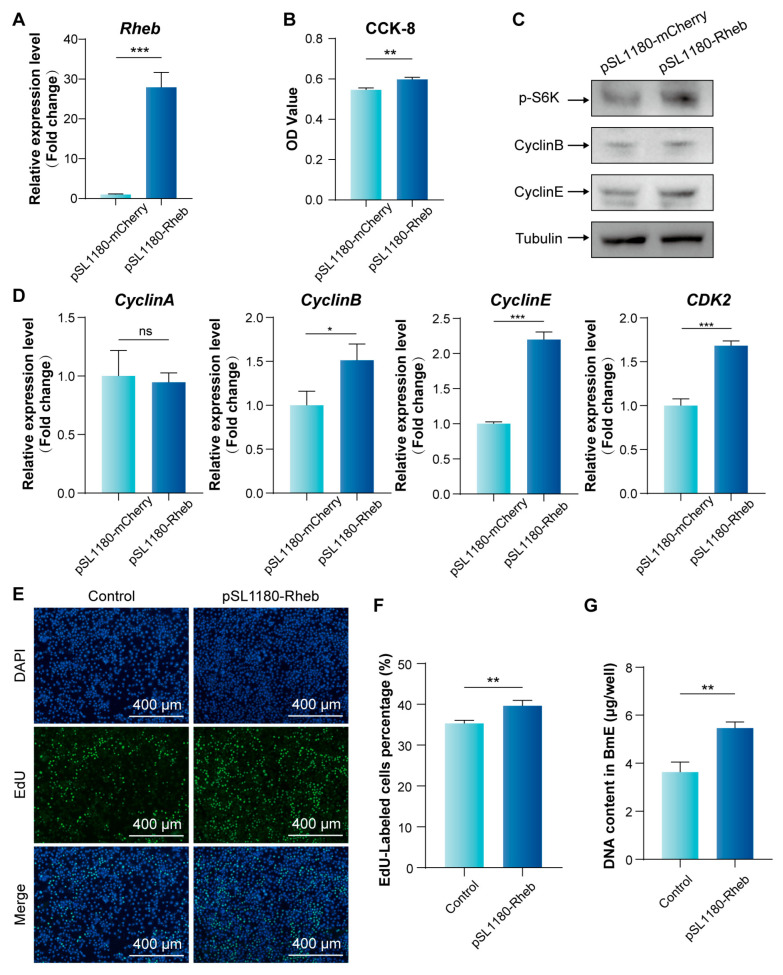
Effect of enhanced mTORC1 signaling pathway activity on BmE cell cycle and cell proliferation. (**A**) qPCR analysis of *Rheb* expression in BmE cells after 48 h post-transfection. (**B**) CCK-8 assay for cell proliferation viability. Error bars indicate SEM (n = 6). (**C**) WB analysis of the phosphorylation level of S6K and the expression of CyclinB and CyclinE protein in BmE cells. (**D**) qPCR analysis of the expression changes in cell cycle-related genes in BmE cells. Error bars indicate SEM (n = 3). (**E**) Effect of *Rheb* overexpression on cell proliferation of BmE cells. Proliferating cells were labeled with EdU (green fluorescence). Cell nuclei were stained with DAPI (blue fluorescence). Scale bar is 400 μm. (**F**) Statistical analysis of the proportion of EdU-positive cells. Cell numbers were calculated from three randomized regions. (**G**) Overexpression of *Rheb* leads to increased DNA content. Error bars indicate SEM (n = 3). Statistically significant differences were assessed using Student’s *t*-test (ns > 0.05, * *p* < 0.05, ** *p* < 0.01, and *** *p* < 0.001).

Our previous studies found that *Rheb* activated TORC1 signaling to accelerate silk protein synthesis in silkworm [26]. Therefore, we further analyzed the effect of enhancing mTORC1 signaling pathway activity on BmE cells. The overexpression of *Rheb* vector (pSL1180-Rheb) was constructed and transfected into BmE cells. The qPCR result indicated significantly higher expression levels of *Rheb* in cells transfected with the overexpression of *Rheb* vector than in the control group (Figure 4A). The up-regulation of *Rheb* was associated with a marked increase in cell proliferative viability (Figure 4B) and enhanced phosphorylation levels S6K (Figure 4C). These data suggest that *Rheb* overexpression enhances mTORC1 activity and promotes cell proliferation.

Further analysis revealed that *Rheb* overexpression significantly up-regulated the expression of *CyclinB*, *CyclinE*, and *CDK2*, with a corresponding increase in the protein levels of CyclinB and CyclinE (Figure 4C,D). To investigate the effect of increased mTORC1 activity on S phase DNA replication, EdU assays demonstrated a rise in the proportion of fluorescently labeled cells to 40.13% (Figure 4E,F), along with a notable augmentation in DNA content (Figure 4G). These results indicate that overexpression of *Rheb* activates the mTORC1 signaling pathway, thereby promoting the expression of key cell cycle genes and proteins in BmE cells and enhancing proliferation, particularly during the S phase of DNA replication.

### 3.4. Inhibition of mTORC1 Signaling Pathway Suppresses Endoreplication in Silk Gland

**Figure 5:**
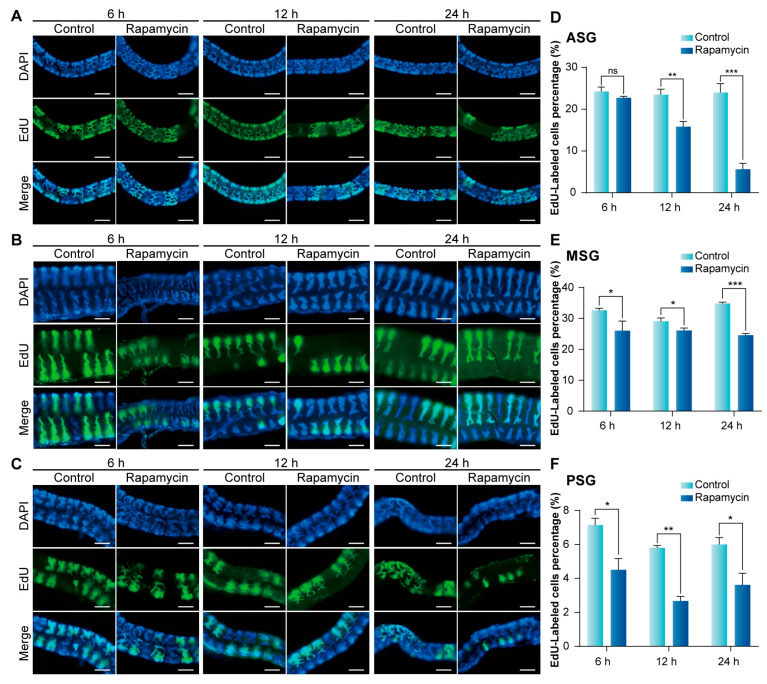
Rapamycin treatment inhibits endoreplication in silkworm silk glands. (**A**) Effect of Rapamycin treatment at different times on endoreplication in ASG of L4D2. Scale bar is 100 μm. (**B**) Effect of Rapamycin treatment at different times on endoreplication in MSG of L4D2. Scale bar is 100 μm. (**C**) Effect of Rapamycin treatment at different times on endoreplication in PSG of L4D2. Scale bar is 100 μm. (**D**–**F**) Statistical analysis of the proportion of EdU-positive cells. ASG, anterior silk gland; MSG, middle silk gland; PSG, posterior silk gland. Statistically significant differences were assessed using Student’s *t*-test (ns > 0.05, * *p* < 0.05, ** *p* < 0.01, and *** *p* < 0.001).

**Figure 6:**
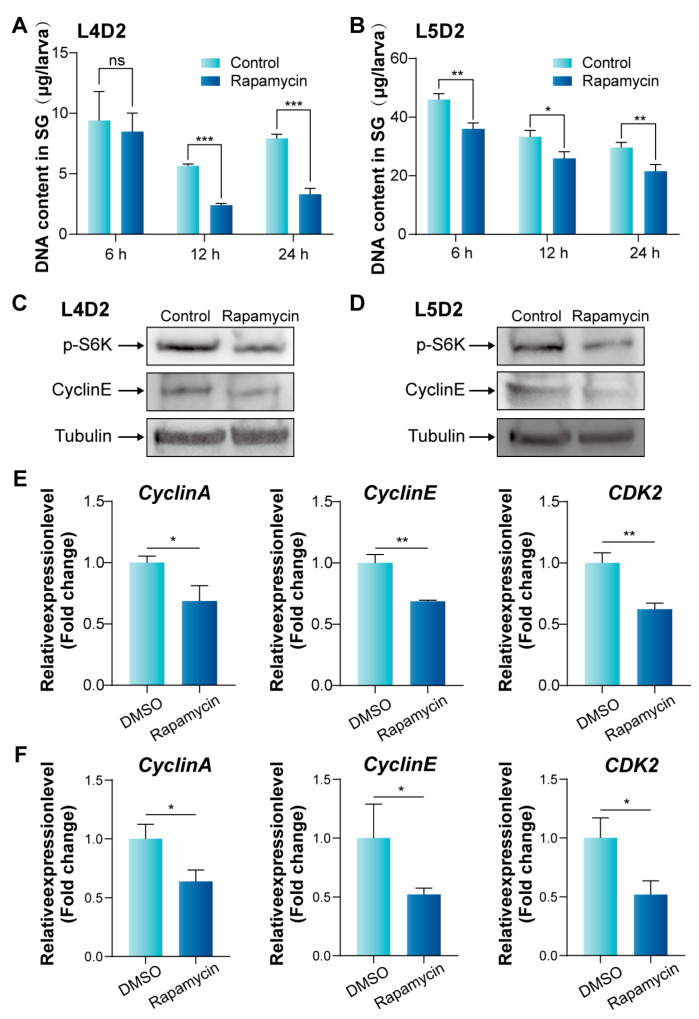
Reduced activity of the mTORC1 signaling pathway inhibits silk gland DNA replication and expression of cell cycle-related gene proteins. (**A**) Reduced activity of the mTORC1 signaling pathway results in reduced DNA content in silk gland of L4D2. Error bars indicate SEM (n = 3). (**B**) Reduced activity of the mTORC1 signaling pathway results in reduced DNA content in silk gland of L5D2. Error bars indicate SEM (n = 3). (**C**,**D**) Western blots depicting the level of phosphorylation of S6K and CyclinE after Rapamycin treatment. (**E**) qPCR analysis of endoreplication-related genes in the silk glands of L4D2 after 12 h of Rapamycin treatment. Error bars indicate SEM (n = 3). (**F**) qPCR analysis of endoreplication-related genes in the silk glands of L5D2 after 6 h of Rapamycin treatment. Error bars indicate SEM (n = 3). Statistically significant differences were assessed using Student’s *t*-test (ns > 0.05, * *p* < 0.05, ** *p* < 0.01, and *** *p* < 0.001).

To verify the effect of the mTORC signaling pathway on BmE cells, we further carried out related experiments on silkworm silk glands of 4th instar larvae at day 2 (L4D2) cultured in vitro. EdU assays showed that the number of EdU-labeled cells in the anterior silk gland (ASG) began to decline after 12 h of Rapamycin treatment (Figure 5A), while reductions in the middle silk gland (MSG) and PSG were observed after 6 h (Figure 5B,C). Counting the EdU-labeled cells confirmed consistency with these observations (Figure 5D–F). Furthermore, we assessed the DNA content in silk glands treated with Rapamycin. A significant decline in DNA content was detected in the silk glands of L4D2 after 12 h of Rapamycin treatment (Figure 6A) and in those of 5th instar larvae at day 2 (L5D2) after 6 h of treatment (Figure 6B). These findings suggest that mTORC1 signaling pathway inhibition suppresses the endoreplication process in the silk glands.

We further characterized the molecular changes in silk gland replication after Rapamycin treatment using WB and qPCR. WB analysis showed that Rapamycin treatment reduced the phosphorylation level of S6K in the silk glands, indicating that the mTORC1 signaling activity in the silk glands was inhibited (Figure 6C,D). qPCR analysis demonstrated that the expression levels of *Cyclin A*, *Cyclin E*, and *CDK2* in the silk glands were significantly down-regulated following Rapamycin treatment (Figure 6E,F). Additionally, WB analysis confirmed a decrease in CyclinE protein levels in the silk glands of both L4D2 and L5D2 following Rapamycin treatment (Figure 6C,D). These findings suggest that inhibiting mTORC1 activity suppresses DNA replication signals in the silk gland cells, leading to a decrease in DNA content and down-regulation of S phase related cell cycle genes, ultimately inhibiting endoreplication in silk gland cells.

### 3.5. Rheb Promotes Endoreplication in PSG and Improves Silk Yield by Enhancing mTORC1 Signaling Pathway Activity

**Figure 7:**
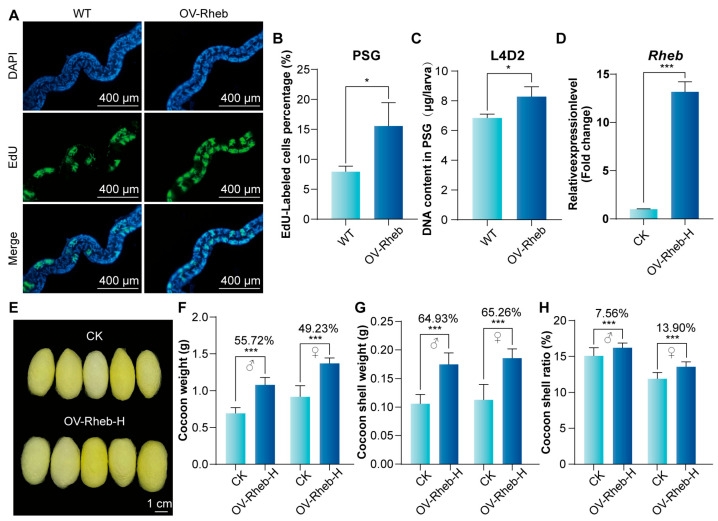
Effect of *Rheb* overexpression on silk gland endoreplication and silk yield (**A**) Edu assay was used to analyze the endoreplication in silk glands. Scale bar is 400 μm. (**B**) Statistical analysis of the proportion of EdU-positive cells. (**C**) Analysis of DNA Content in the PSG at L4D2. Error bars indicate SEM (n = 3). (**D**) qPCR analysis of *Rheb* expression. CK: Offspring of female moths D9L crossed with male moths *Chuanshan*; OV-Rheb-H: Offspring of female moths OV-Rheb crossed with male moths *Chuanshan*. (**E**) Morphological observations of control and experimental group cocoons. (**F**–**H**) Statistical analysis of cocoon weight, cocoon shell weight, and cocoon layer ratio. (n = 30). Statistically significant differences were assessed using Student’s *t*-test (* *p* < 0.05, *** *p* < 0.001).

Using the PSG-specifically overexpressed *Rheb* silkworm (OV-Rheb), which was established in our previous studies [26], we investigated the impact of *Rheb* on endoreplication in PSG cells. The results of cell proliferation assay showed that the number of EdU-labeled cells in PSG of OV-Rheb silkworm was significantly higher than WT (Figure 7A,B). This indicates that Rheb promotes the replication of DNA in the PSG, which is consistent with the increase in DNA content (Figure 7C).

The endoreplication of silk gland cells is closely related to the expression of silk proteins and silk yield [16,26,32]. To further verify the potential of the mTORC1 signaling pathway in improving the economic traits of silkworms, we conducted a crossbreeding experiment between female moths overexpressing Rheb specifically in the PSG of OV-Rheb and male moths of the *Chuanshan* strain, which is widely used in sericulture. After hybridization, qPCR analysis showed that the overexpression trait of *Rheb* could be transferred to the offspring, designated as OV-Rheb-H. Notably, the cocoons produced by these individuals were significantly larger compared to those of the control group (Figure 7D,E). Furthermore, cocoon yield performance traits were investigated and analyzed. The cocoon weight of OV-Rheb-H increased by about 55.72% in males and 49.23% in females (Figure 7F); while the cocoon shell weight increased significantly by 64.93% and 65.26% in males and females, respectively (Figure 7G). Similarly, the cocoon layer rate of OV-Rheb-H in both males and females has also been significantly improved (Figure 7H).

## 4. Discussion

The mTORC1 signaling pathway, present across a wide range of organisms, is responsible for detecting and reacting to diverse nutritional and environmental factors such as growth factors, energy levels, cellular stress, and amino acids [20,33,34]. Upon activation, mTORC1 interacts with numerous downstream substrates, playing a critical role in anabolic and catabolic processes, inhibiting autophagy and lysosomal biogenesis, promoting the synthesis of proteins, nucleotides, lipids, and other macromolecules [35]. These actions collectively regulate essential processes of cellular growth and development [36,37]. While the mTORC1 signaling pathway serves as a central hub for numerous cellular signaling networks, research has predominantly focused on its regulatory mechanisms in human diseases and cancers, with comparatively fewer studies exploring its role in regulating insect physiology. It is imperative to conduct further research that elucidates the role of mTORC1 within the regulatory framework of insect growth and development.

Rapamycin, a potent and specific inhibitor of mTOR, binds to FKBP12, thereby inhibiting mTORC1 activity [38,39]. In *Drosophila*, the deletion of *dTOR* induces a cellular phenotype that resembles amino acid deprivation, marked by reduced nucleolus size, lipid vesicle aggregation in larval fat bodies, and cell cycle arrest in a cell type-specific manner [40,41]. In this study, we established that the optimal Rapamycin treatment for BmE cells is 10 μM for 12 h. The treatment with Rapamycin inhibited mTORC1 signaling in BmE cells, resulting in decreased expression of CyclinB and CyclinE proteins, reduced DNA replication rates, and cell cycle arrest at the G2/M phase. At the silk gland level, Rapamycin treatment of isolated cultured glands inhibited mTORC1 signaling, leading to reduced DNA content due to impaired DNA replication. Furthermore, the expression of *CyclinA*, *CyclinE*, and *CDK2*, which are essential for maintaining the S phase of endoreplication in the silk gland, was significantly down-regulated.

Activation of the mTORC1 signaling pathway in *Drosophila* promotes the growth of eye, head, and wing cells, influencing cell cycle genes and regulatory factors [27,42]. Overexpression of *dRheb* at the organismal level leads to tissue overgrowth and increased cell size throughout the organism, while overexpression at the cellular level results in the accumulation of cells in the S phase [29,43,44]. Conversely, reduced *dRheb* activity causes decreased tissue growth and smaller cell size, as well as G1 phase arrest and reduced cell size in cultured cells [45]. In our previous study, the overexpression of *Rheb* in PSG led to the increased synthesis of silk proteins and enlarged silk gland organs, ultimately enhancing silk production and cocoon shell rate. In the present study, our findings suggest that the enhancement of mTORC1 signaling pathway signals leads to strengthened endoreplication, accelerating the rate of S phase DNA replication synthesis, continuous accumulation of genetic material, and improved protein translation efficiency.

Based on previous studies and our results, we suggest that the mTORC1 signaling pathway promotes the expression of silk protein related genes and the enlargement of silk glands by enhancing the endoreplication efficiency of silk gland cells, which ultimately leads to the increase in silk production. These findings enrich the regulatory network of endoreplication in insects and provide new insights for further studies on the role of the mTORC1 signaling pathway in silk gland development. Furthermore, this work provides materials and insights for breeding high-yield silkworm varieties, thereby contributing to the advancement of breeding techniques in the sericulture industry.

## Data Availability

The original contributions presented in this study are included in the article/Supplementary Materials. Further inquiries can be directed to the corresponding author.

## Supplementary Materials

The following supporting information can be downloaded at: https://www.mdpi.com/article/10.3390/insects16070647/s1, Table S1: Primer sequences, Figure S1: Original Western blot images for Figure 1C, Figure S2: Original Western blot images for Figure 2B, Figure S3: Original Western blot images for Figure 4C, Figure S4: Original Western blot images for Figure 6C, Figure S5: Original Western blot images for Figure 6D.
