# Supplementary figures and images for: The Rheb-mTORC1 Coordinates Cell Cycle Progression and Endoreplication in Bombyx mori
Source: Insects. 2025 Jun 20;16(7):647. doi: 10.3390/insects16070647 (PMC12295899; doi:10.3390/insects16070647)

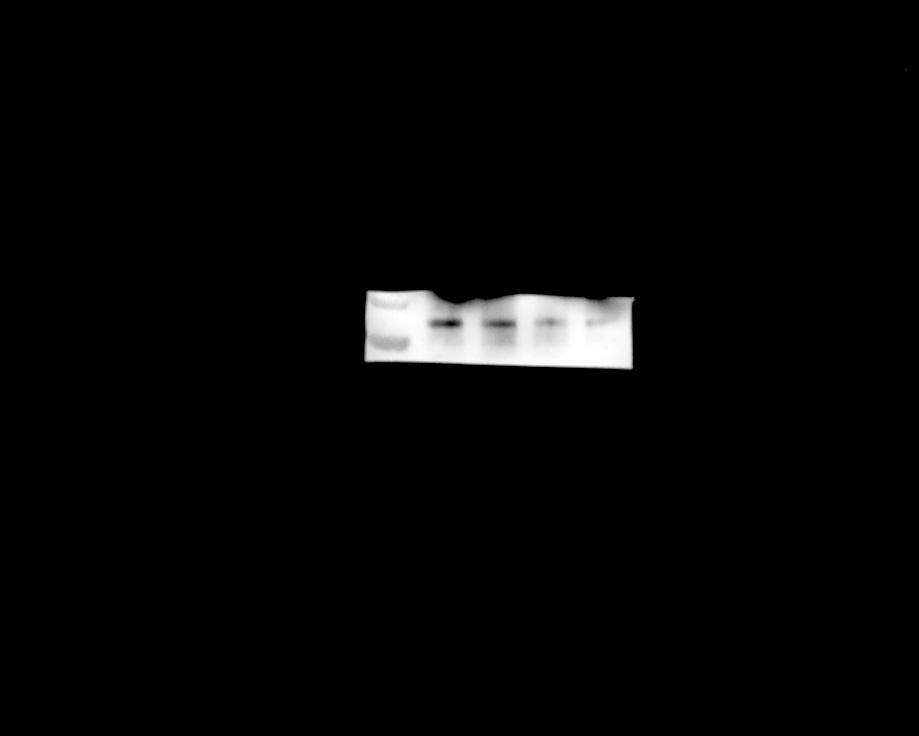

Supplement: Supplementary file 1 [file insects-16-00647-s001.zip › Figure S1/p-S6K.jpg]

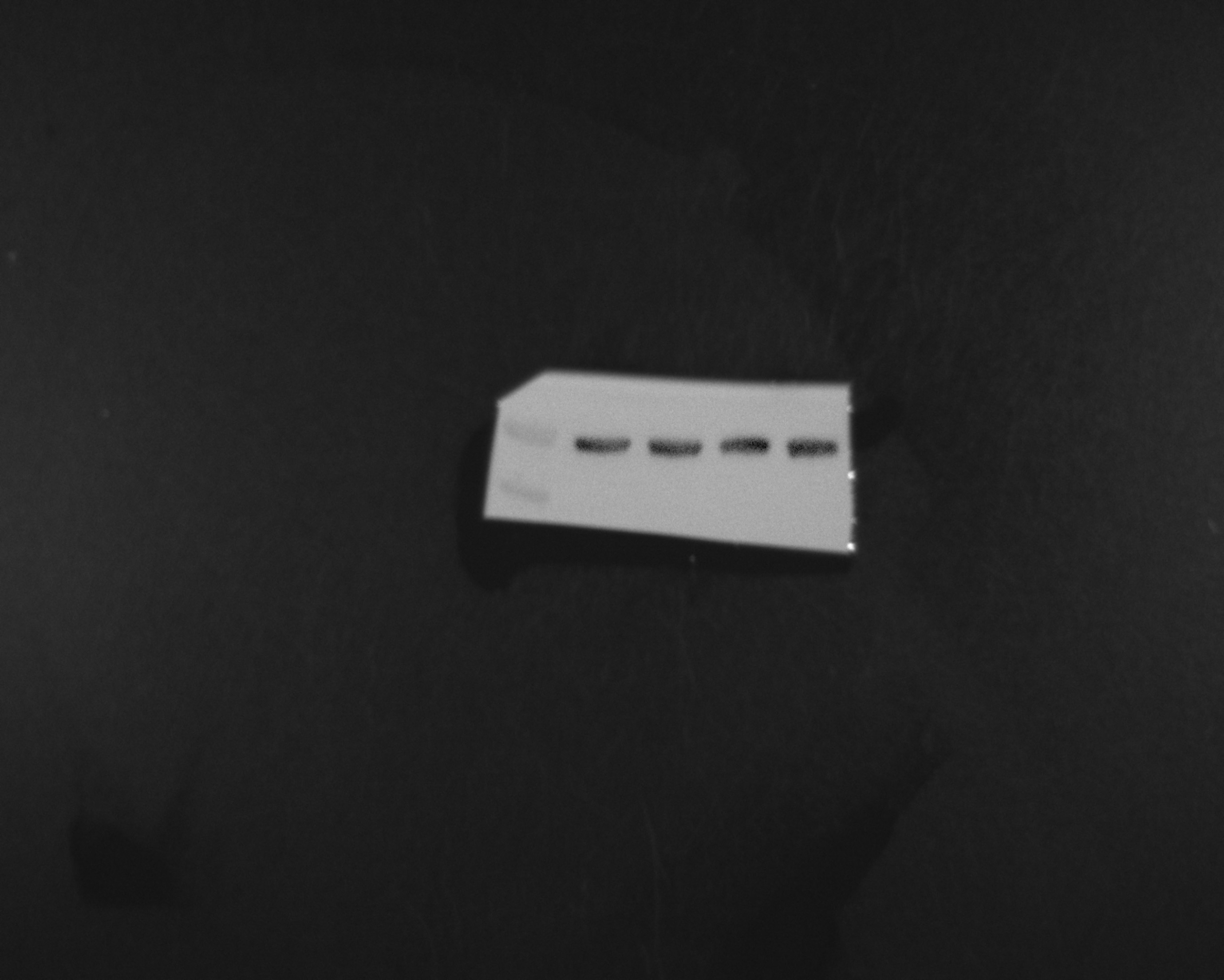

Supplement: Supplementary file 1 [file insects-16-00647-s001.zip › Figure S1/Tubulin.jpg]

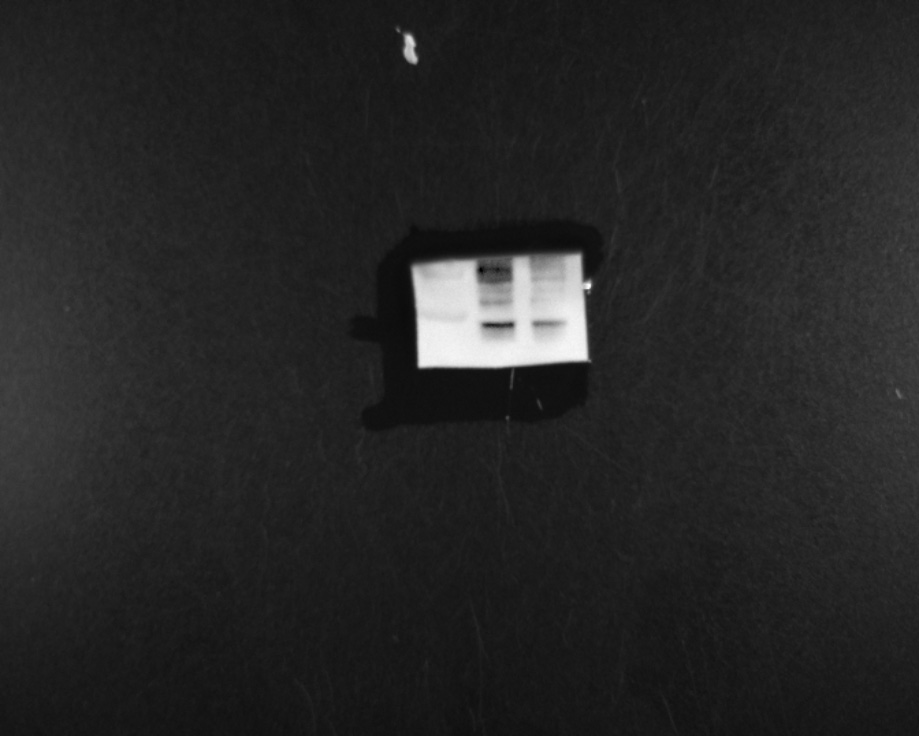

Supplement: Supplementary file 1 [file insects-16-00647-s001.zip › Figure S2/CyclinB.jpg]

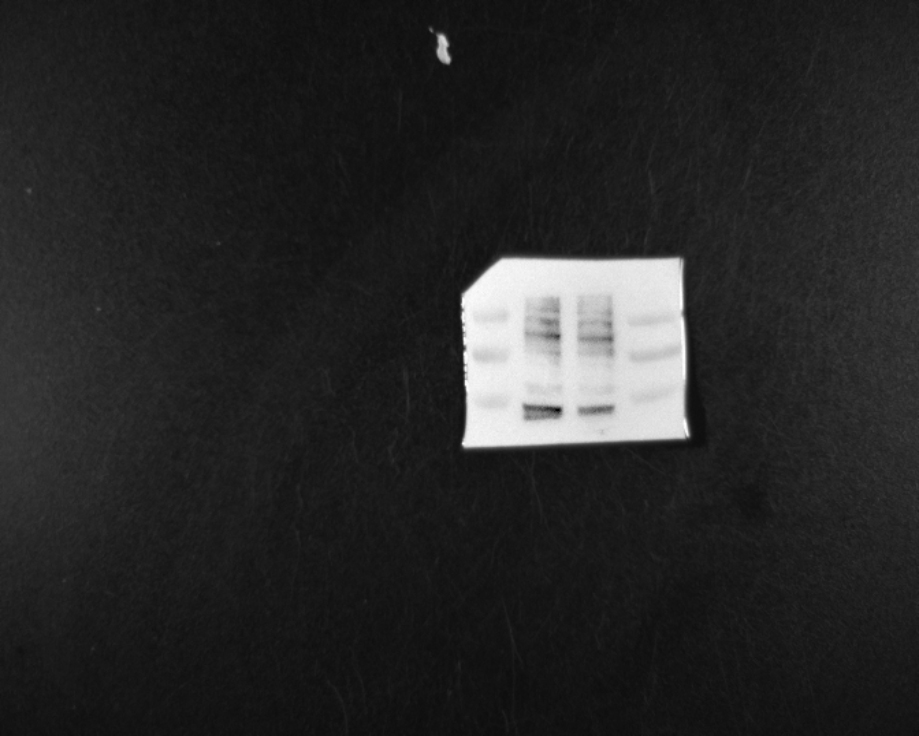

Supplement: Supplementary file 1 [file insects-16-00647-s001.zip › Figure S2/CyclinE.jpg]

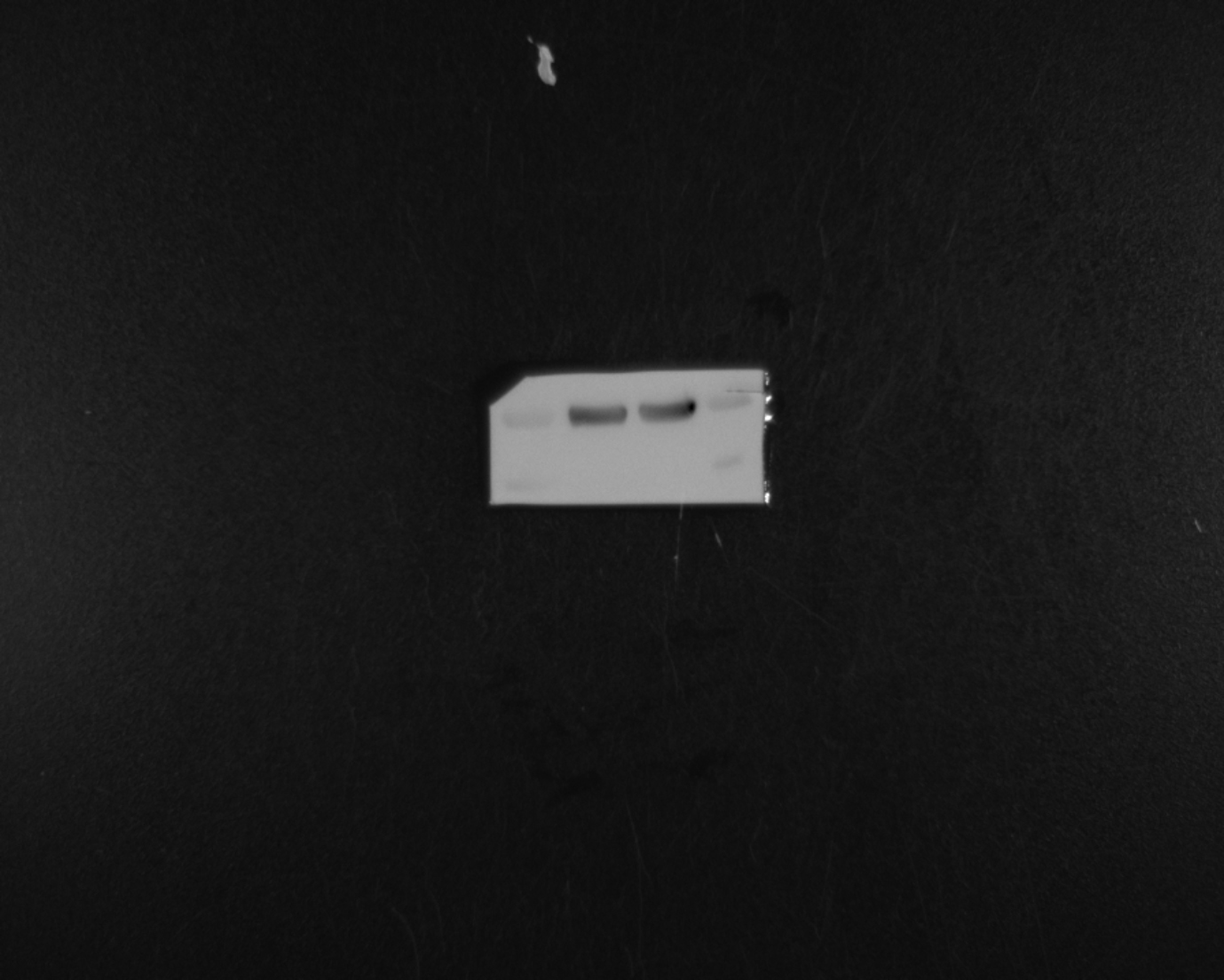

Supplement: Supplementary file 1 [file insects-16-00647-s001.zip › Figure S2/Tubulin.jpg]

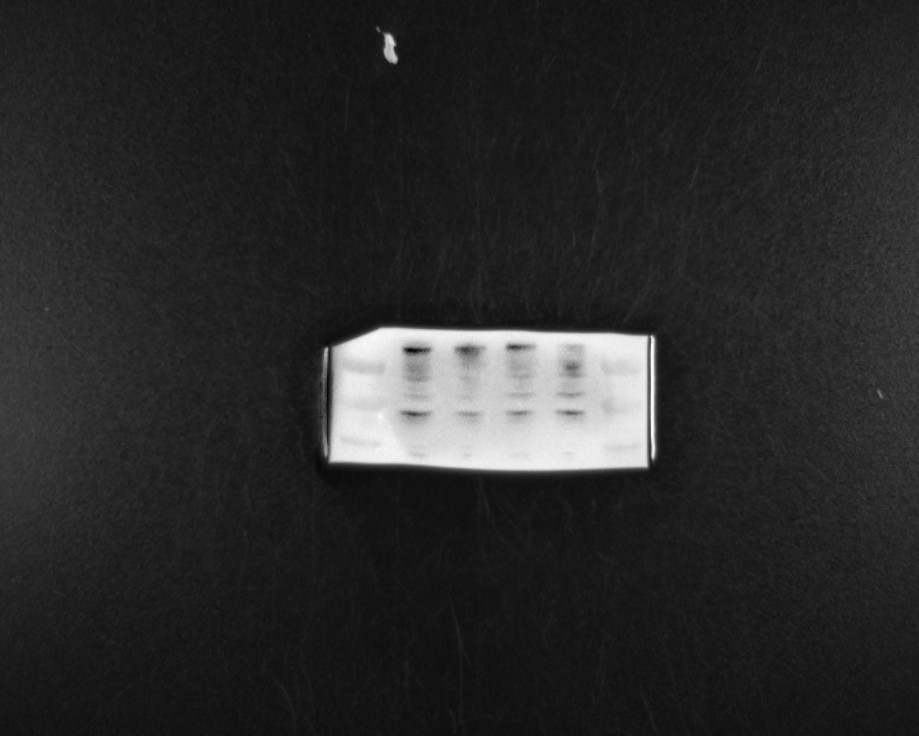

Supplement: Supplementary file 1 [file insects-16-00647-s001.zip › Figure S3/CyclinB.jpg]

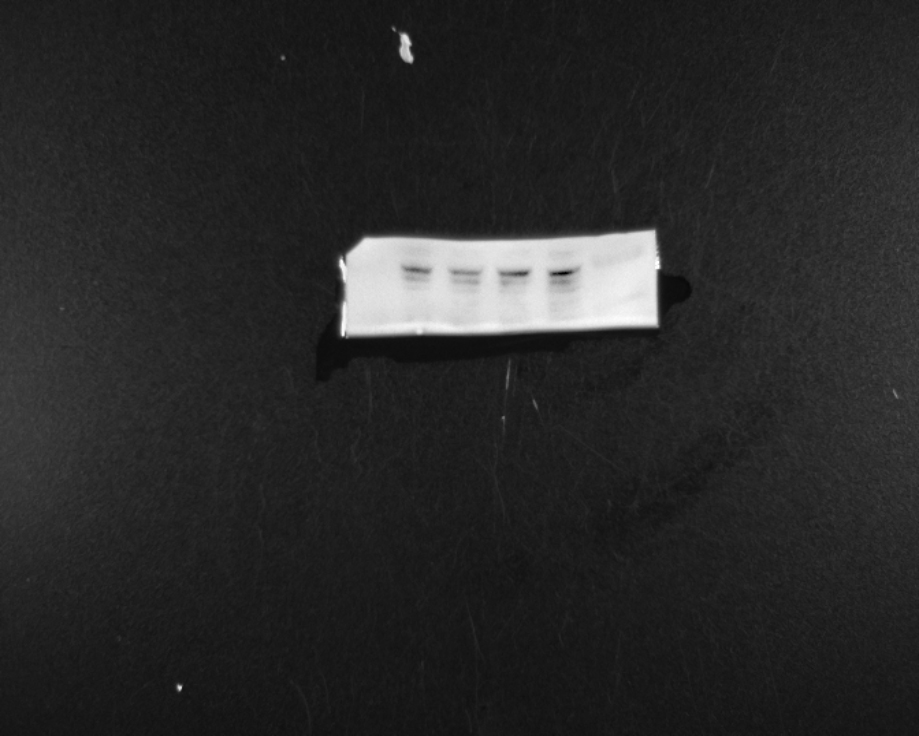

Supplement: Supplementary file 1 [file insects-16-00647-s001.zip › Figure S3/CyclinE.jpg]

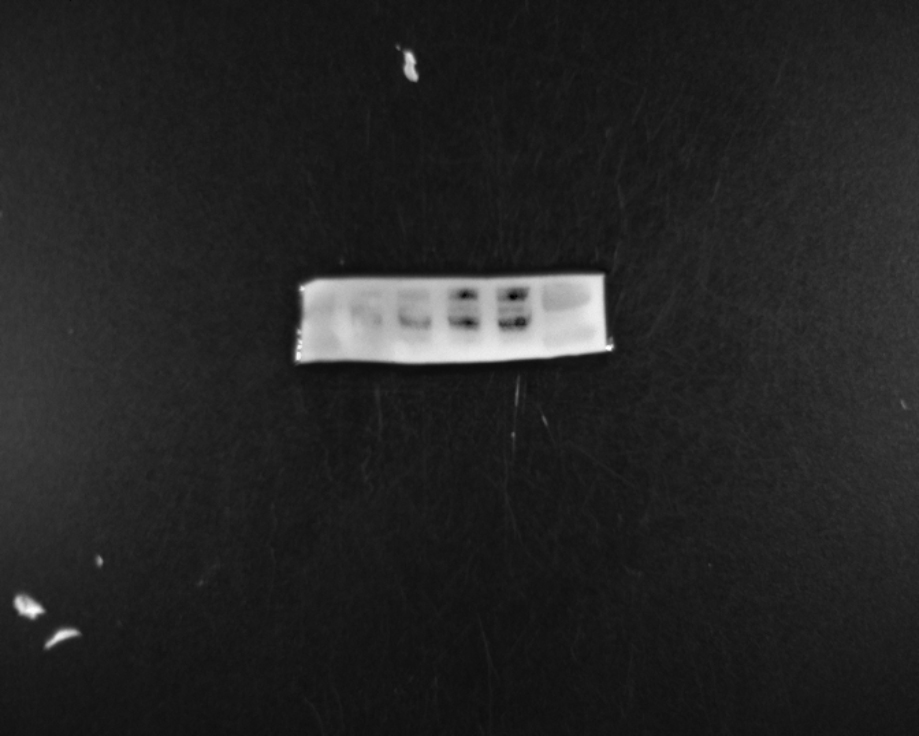

Supplement: Supplementary file 1 [file insects-16-00647-s001.zip › Figure S3/p-S6K.jpg]

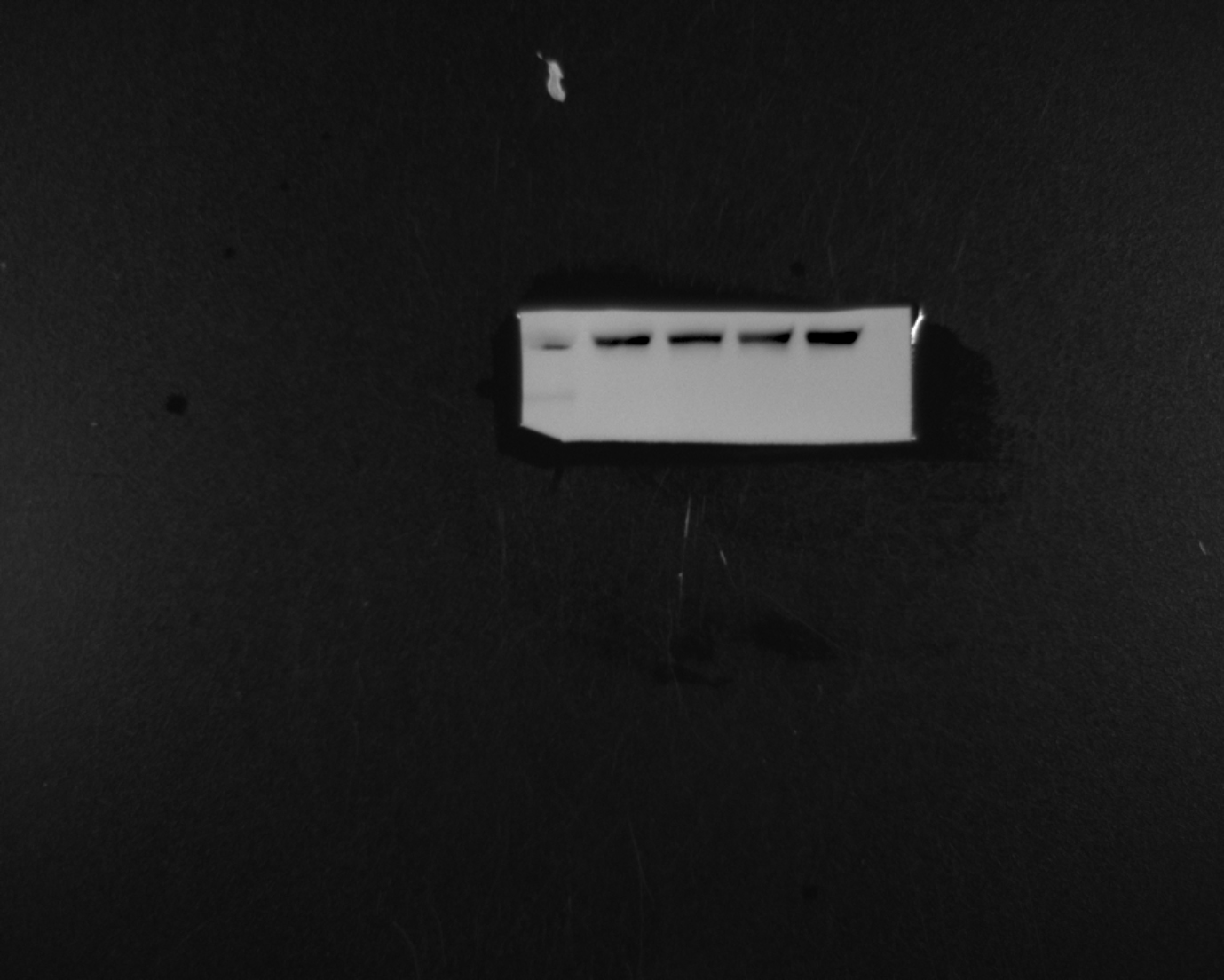

Supplement: Supplementary file 1 [file insects-16-00647-s001.zip › Figure S3/Tubulin.jpg]

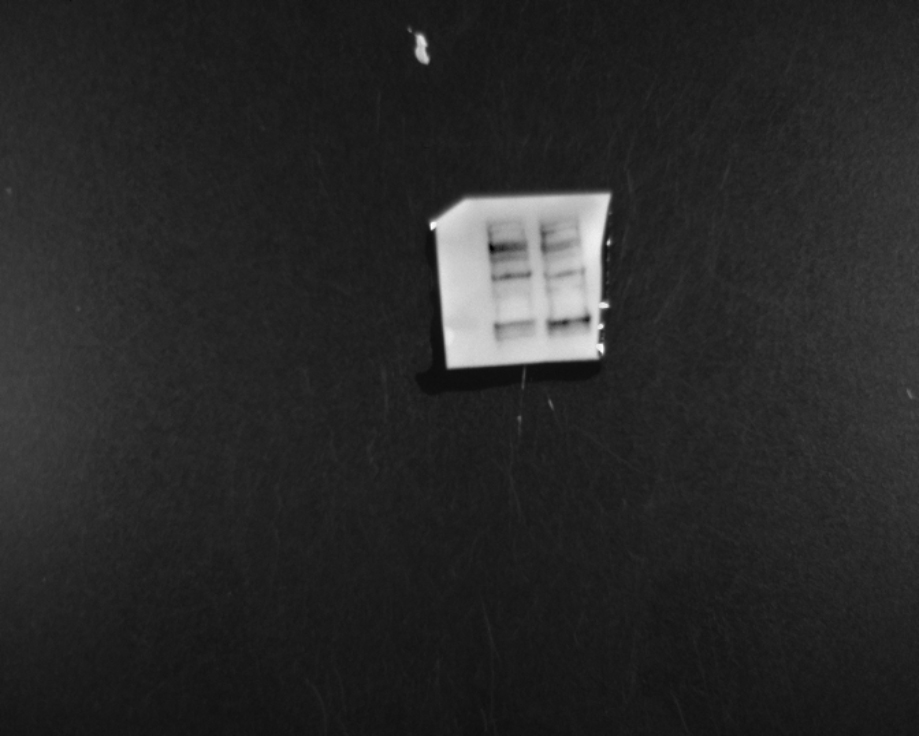

Supplement: Supplementary file 1 [file insects-16-00647-s001.zip › Figure S4/CyclinE.jpg]

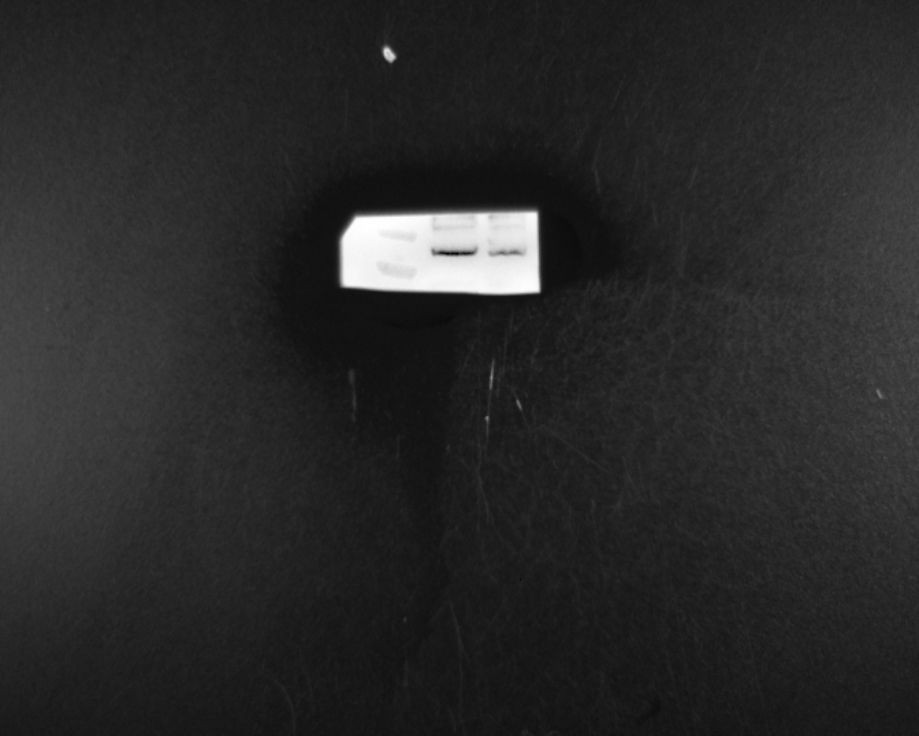

Supplement: Supplementary file 1 [file insects-16-00647-s001.zip › Figure S4/p-S6K.jpg]

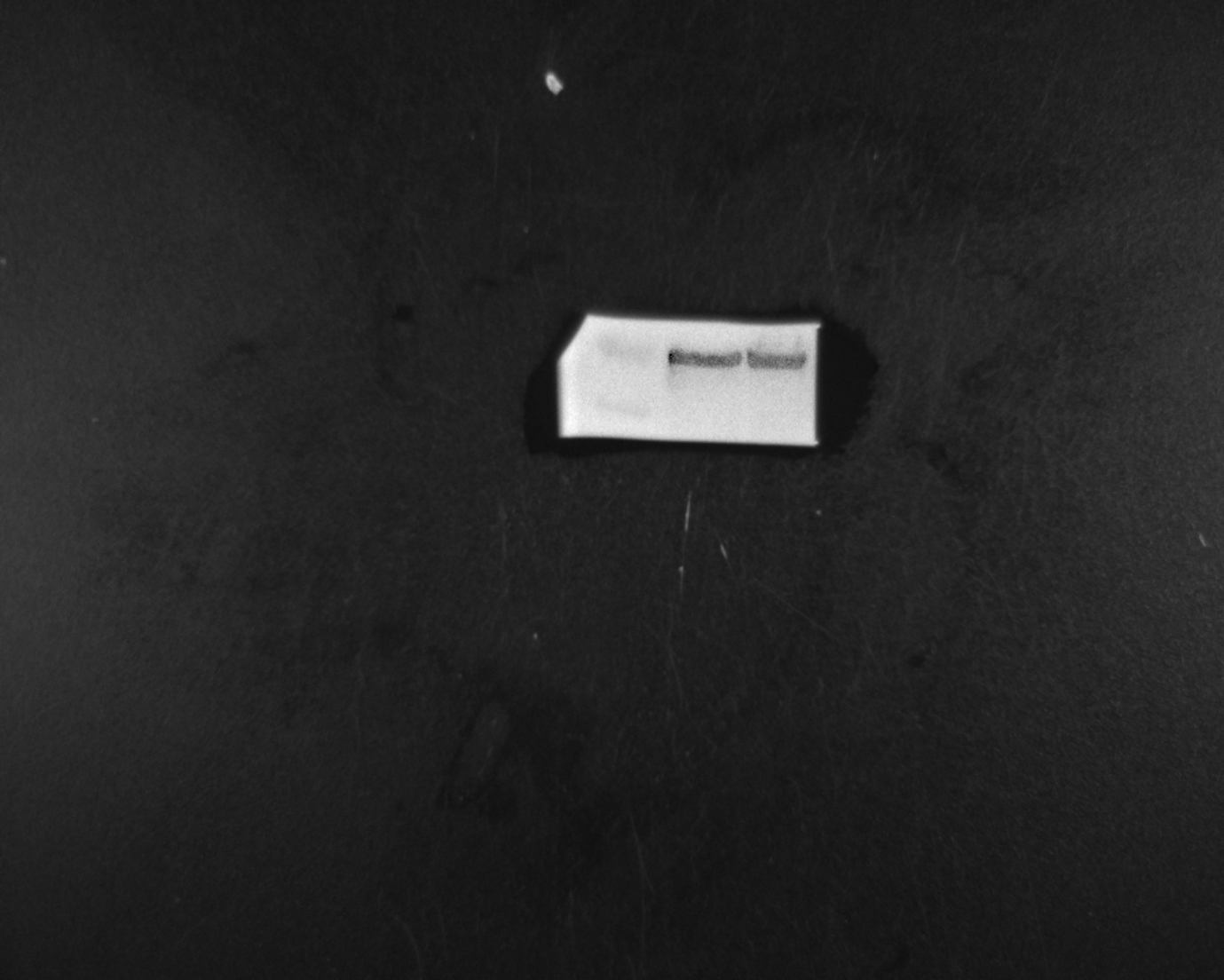

Supplement: Supplementary file 1 [file insects-16-00647-s001.zip › Figure S4/Tubulin.jpg]

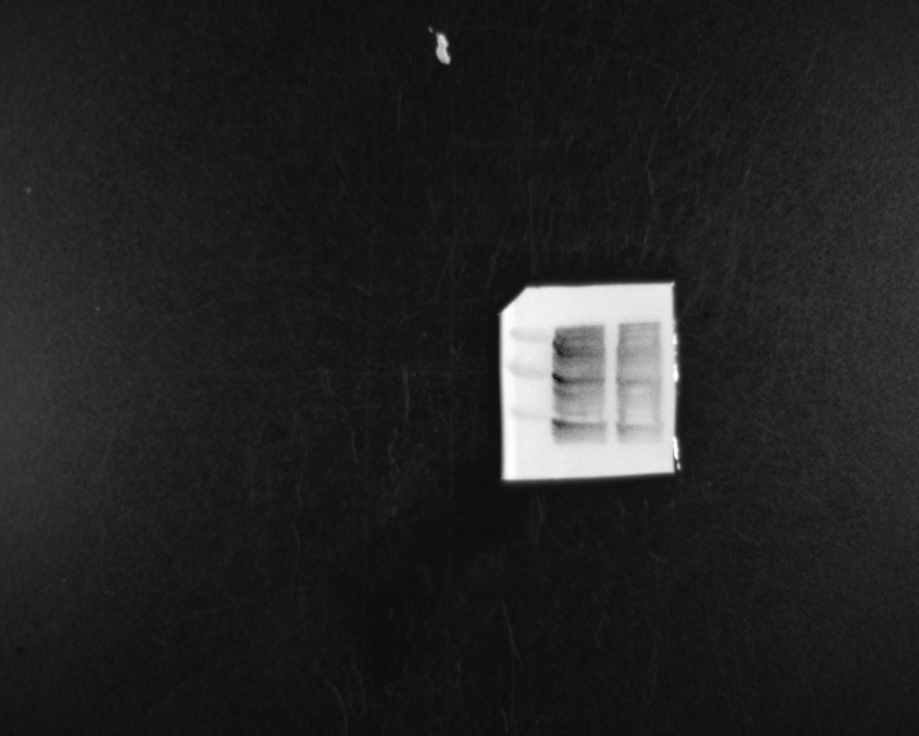

Supplement: Supplementary file 1 [file insects-16-00647-s001.zip › Figure S5/CyclinE.jpg]

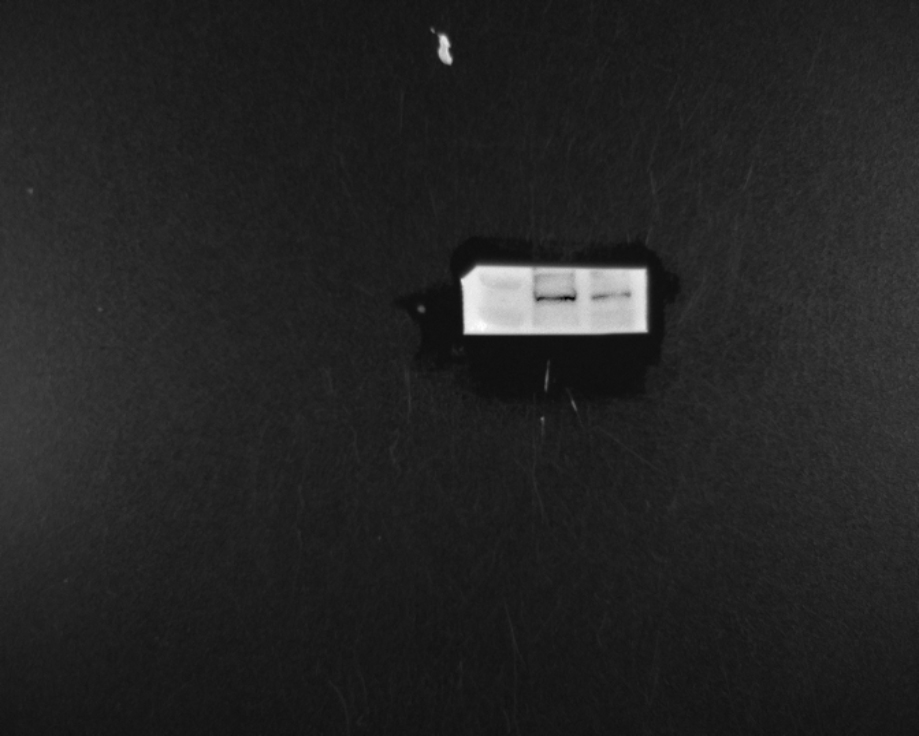

Supplement: Supplementary file 1 [file insects-16-00647-s001.zip › Figure S5/p-S6K.jpg]

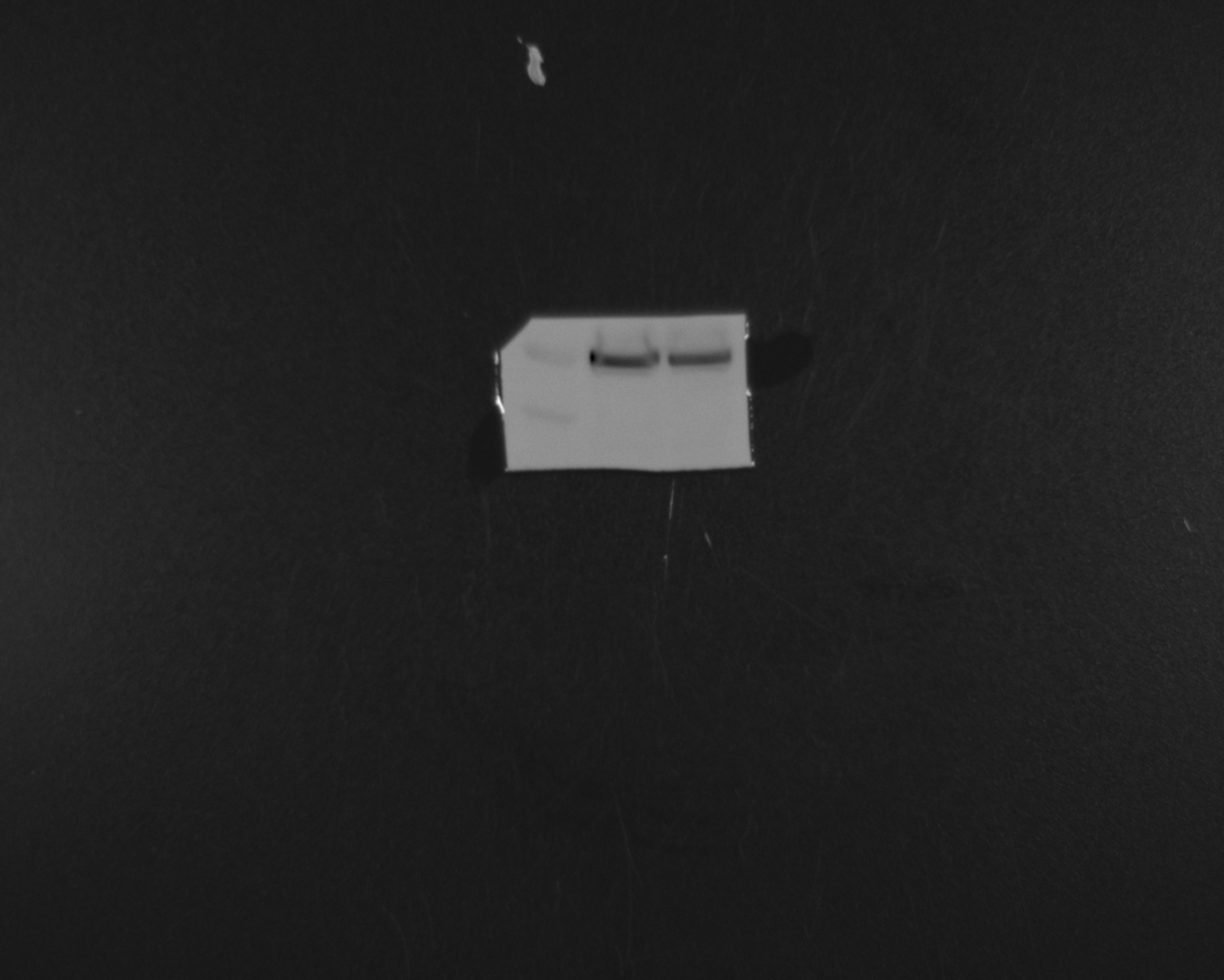

Supplement: Supplementary file 1 [file insects-16-00647-s001.zip › Figure S5/Tubulin.jpg]
